# Cord serum cytokines at birth and children's trajectories of mood dysregulation symptoms from 3 to 8 years: The EDEN birth cohort

**DOI:** 10.1016/j.bbih.2024.100768

**Published:** 2024-03-29

**Authors:** Marie Herbein, Susana Barbosa, Ophélie Collet, Olfa Khalfallah, Marie Navarro, Marion Bailhache, Nicolas IV, Bruno Aouizerate, Anne-Laure Sutter-Dallay, Muriel Koehl, Lucile Capuron, Pierre Ellul, Hugo Peyre, Judith Van der Waerden, Maria Melchior, Sylvana Côté, Barbara Heude, Nicolas Glaichenhaus, Laetitia Davidovic, Cedric Galera

**Affiliations:** a University of Bordeaux, France; b INSERM, Bordeaux Population Health Center, UMR1219, France; c Centre Hospitalier Perrens, Bordeaux, France; d Université Côte d’Azur, Nice, France; e Centre National de Recherche Scientifique, Institut de Pharmacologie Moléculaire et Cellulaire, Valbonne, France; f Research Unit on Children's Psychosocial Maladjustment, Montreal, Canada; g Centre Hospitalier Universitaire de Bordeaux, Département de Pédiatrie, France; h INRAE, Bordeaux INP, NutriNeuro, UMR 1286, Bordeaux, France; i INSERM, Neurocentre Magendie, UMR1215, Bordeaux, France; j Robert Debré Hospital, Child and Adolescent department, APHP, Paris University, Paris, France; k Immunology-Immunopathology-Immunotherapy (i3), UMRS 959, INSERM, Paris, France; l Centre de Ressource Autisme Languedoc-Roussillon et Centre d'Excellence sur l'Autisme et les Troubles du Neurodéveloppement (CeAND), CHU Montpellier, Montpellier, France; m Université Paris Saclay, UVSQ, Inserm, CESP, Tem DevPsy, 94807, Villejuif, France; n INSERM U1136, Institut Pierre Louis d’Epidémiologie et de Santé Publique, Sorbonne Université, Équipe de Recherche en Épidémiologie Sociale, Paris, France; o University of Montreal, Department of Social and Preventive Medicine, Montreal, Canada; p Université de Paris, Centre for Research in Epidemiology and Statistics (CRESS), INSERM, INRAE, F-75004, Paris, France; q Paris University, France; r Fondation FondaMental, Créteil, France

**Keywords:** Irritability, DMDD, Immune system, Cytokines, Child and adolescent psychiatry, TNFα, Pregnancy, Epidemiology, Birth cohort

## Abstract

There is growing evidence that *in utero* imbalance immune activity plays a role in the development of neurodevelopmental and psychiatric disorders in children. Mood dysregulation (MD) is a debilitating transnosographic syndrome whose underlying pathophysiological mechanisms could be revealed by studying its biomarkers using the Research Domain Criteria (RDoC) model. Our aim was to study the association between the network of cord serum cytokines, and mood dysregulation trajectories in offsprings between 3 and 8 years of age. We used the data of a study nested in the French birth cohort EDEN that took place from 2003 to 2014 and followed mother-child dyads from the second trimester of pregnancy until the children were 8 years of age. The 2002 mother-child dyads were recruited from the general population through their pregnancy follow-up in two French university hospitals. 871 of them were included in the nested cohort and cord serum cytokine levels were measured at birth. Children's mood dysregulation symptoms were assessed with the Strengths and Difficulties Questionnaire Dysregulation Profile at the ages 3, 5 and 8 years in order to model their mood dysregulation trajectories. Out of the 871 participating dyads, 53% of the children were male. 2.1% of the children presented a high mood dysregulation trajectory whereas the others were considered as physiological variations. We found a significant negative association between TNF-α cord serum levels and a high mood dysregulation trajectory when considering confounding factors such as maternal depression during pregnancy (adjusted Odds Ratio (aOR) = 0.35, 95% Confidence Interval (CI) [0.18–0.67]). Immune imbalance at birth could play a role in the onset of mood dysregulation symptoms. Our findings throw new light on putative immune mechanisms implicated in the development of mood dysregulation and should lead to future animal and epidemiological studies.

## Highlights

• A small group of children follows a high mood dysregulation trajectory early on.
• A complex network of cord blood cytokines is associated with child mood dysregulation.
• Low TNF alpha in cord blood is specifically related to child mood dysregulation.

## Abbreviations:

ADHD: Attention Deficit Hyperactivity Disorder
aOR: adjusted Odds Ratio
ASD: Autism Spectrum Disorder
BMI: Body Mass Index
CCL: Chemokine (C-Cmotif) ligand
CES-D: Center of Epidemiological Studies Depression Scale
CI: Confidence Interval
CXCL: C-X-C motif chemokine
DAG: Directed Acyclic Graph
EDEN: Étude sur les Déterminants pré-et postnatals précoces du développement psychomoteur et de la santé de l’Enfant
GM-CSF: Granulocyte-Macrophage Colony-Stimulating
IFN: Interferon
IL: Interleukin
MD: Mood Dysregulation
MIA: Maternal Immune Activation
NPC: Neural progenitor cells
RDoC: Research Domain Criteria
SDQ: Strengths and Difficulties Questionnaire
SDQ-DP: Strengths and Difficulties Questionnaire- Dysregulation Profile
TNF: Tumor Necrosis Factor
VEGF: Vascular Endothelial Growth Factor
VIP: Variable Inclusion Probability
Lcmm: Latent class mixed model
GBTM: Grouped Based Trajectories Modeling

## 1 Introduction

There is increasing evidence that the immune system and specifically cytokines play a significant role in normal brain development and homeostatic function, and therefore in the early development of psychiatric disorders. For example, in adolescents at risk of developing a depressive episode, only those with higher blood IL-6 levels will develop affective disorder later on (Miller and Cole, 2012). Thus, adolescents presenting with anxiety and depression display higher blood levels not only of pro-inflammatory cytokines like interleukin (IL)-1β, IL-6, and interferon (IFN)-γ but also of anti-inflammatory cytokines such as IL-1 (Henje Blom et al., 2012; Gabbay et al., 2009a, 2009b; Howe and Lynch, 2022). In addition, *post mortem* studies have found that adolescents deceased by suicide showed over-expression of the pro-inflammatory cytokines IL-1β and tumor necrosis factor (TNF)-α genes in their prefrontal lobe (Pandey et al., 2012). Other neurodevelopmental conditions in children and adolescents were also associated with changes in blood cytokine levels, with increased IL-13 and IL-16 in attention deficit hyperactivity disorder (ADHD) (Oades et al., 2010) and increased TNF-α and IL-17A in autism spectrum disorder (ASD) compared to neurotypical youths (Siniscalco et al., 2018).

The fetal brain is developing during pregnancy, so it is particularly vulnerable to environmental insults. It has thus been hypothesized that chronic diseases, including mental disorders, might have a developmental origin (Heindel and Vandenberg, 2015; Faa et al., 2016). For example, the risk of neurodevelopmental disorders increases in children exposed *in utero* to maternal immune activation (MIA), due either to gestational infection or to maternal conditions accompanied with immune activation (e.g. stress, depression, obesity, auto-immune diseases) (Grandjean and Landrigan, 2014; Knuesel et al., 2014; Melchior et al., 2015; Polańska et al., 2015; Naudé et al., 2022). Furthermore, low maternal depressive and stress trajectories from pregnancy through offsprings’ adolescents was associated with fewer internalizing and externalizing symptoms than youths whose mothers belonged to an aberrant trajectory (Bailey et al., 2023). This could be related to the secretion of the placental corticotropic hormone which organizes human neurobehavioral development; the exposition to mild stress promoting hippocampal dependent cognitive function whereas severe stress impairs neuronal function and survival (Avishai-Eliner et al., 2002; Sandman, 2015). It has been hypothesized that maternal cytokines play a role in the development of the fetus by crossing the placental barrier, and/or by directly affecting placental function, and/or by modifying placental development and function, and/or by creating fetal immune dysregulation (Woods et al., 2023; Monk et al., 2019).

Cytokines regulate neurodevelopment and brain function in homeostatic conditions (Deverman and Patterson, 2009). The developing brain physiologically expresses cytokines and/or cytokine receptors in spatiotemporally defined patterns, and in neuronal cells (neural progenitor cells (NPC), neurons) and glial cells (astrocytes, microglia) (Deverman and Patterson, 2009). In murine models, the balance between the formation of excitatory/inhibitory synapses is influenced by IL-6 neonatal overexpression (Wei et al., 2012). In addition, hippocampal maturation is accelerated in *Tnf*-knockout pups (Golan et al., 2004), leading to synaptic scaling defects (Stellwagen and Malenka, 2006). In *in vitro* models, the survival, proliferation, and neuronal differentiation of murine NPC is promoted by TNF supplementation (Bernardino et al., 2008). Therefore, animal models have shown that MIA is associated with impaired fetal neurodevelopmental processes including impaired oligodendrocyte growth (Al-Haddad et al., 2019), neurotransmission (Goeden et al., 2016) and synaptic plasticity (Estes and McAllister, 2016), which could lead in turn to cognitive and behavioral abnormalities in the offspring (Monk et al., 2019). In humans, the few epidemiological studies available have confirmed that MIA is linked to ASD onset. Interestingly, schizophrenia has been found to be associated with elevated maternal IL-8 blood concentrations during the second trimester of pregnancy, and anxiety-depression symptoms were related with lower IL-7 in the cord serum at birth (Al-Haddad et al., 2019; Ratnayake et al., 2013; Galera et al., 2021). Furthermore, prenatal exposure, during the third trimester to maternal imbalance of immune activity resulted in dysregulation of the stress circuitry at 45, with sex-dependent effects (Goldstein et al., 2021). Altogether, these data suggest that cytokine signaling around birth impacts neurodevelopment and possibly later behavioral outcome.

The Research Domain Criteria (RDoC) approach, which aims at disentangling the complex origins of psychiatric disorders, is highly relevant for studying their fetal etiology. The RDoC promotes both the use of biomarkers (e.g. cytokines) and transnosographic dimensions to account for the heterogeneity of underlying mechanisms and of interindividual variations in clinical expressions (Sanislow et al., 2019). In children, mood dysregulation (MD) is a highly relevant transnosographic dimension to apply the RdoC model (Meyers et al., 2017; Pacheco et al., 2022). MD, which involves deficits in self-regulation, is a common condition shared by a wide range of neurodevelopmental, emotional, and behavioral disorders covering ADHD, ASD, anxiety, depression, oppositional defiant disorder and conduct disorder (Mikita and Stringaris, 2013; Bruno et al., 2019). It is an observable syndrome that encompasses chronic irritability interspersed with temper tantrums and it often heralds the need for challenging clinical care in child and adolescent psychiatry (Brotman et al., 2017).

The closest disorder to MD in the DSM-5, i.e. disruptive mood dysregulation disorder, has an estimated lifetime prevalence between 0.4 and 3.3% (Carlson and Pataki, 2016). MD can greatly impair a child's functioning during childhood (Dougherty et al., 2016; Copeland et al., 2013; Evans et al., 2017; Mulraney et al., 2016) and can continue into adulthood by increasing the risk of developing affective disorders (Brotman et al., 2017; Evans et al., 2017; Copeland et al., 2015). It is both a serious mental health and public health challenge since it generates a heavy social, educational, and family burden.

To our knowledge, research on MD is limited. First, most current knowledge arises from animal data and human studies are scarce. Second, few studies used cord serum as the matrix to measure cytokines, even though it reflects maternal and fetal immune activity at birth. Third, prior studies generally focused on restricted panels of biomarkers, thereby potentially missing relevant cytokines. Fourth, no study has specifically explored the association between prenatal immune activity and the emergence of MD trajectories during childhood.

Our aim was to study the association between the network of cord serum cytokines at birth and MD trajectories in the offspring between 3 and 8 years of age.

## 2 Methods

### 2.1 The cohort

EDEN (Étude sur les Déterminants pré-et postnatals précoces du développement psychomoteur et de la santé de l’ENfant) is a French birth cohort that recruited mother-child dyads from pregnancy to birth and through childhood to study early determinants of the physical, psychological, and social development of children. Pregnant women were recruited during their medical appointment before their 24th week of amenorrhea between 2003 and 2006 at the Nancy and Poitiers university hospitals. Being a minor, multiple pregnancy, having a history of diabetes, illiteracy or planning to move in the three years to come were exclusion criteria. Two thousand and two women were included out of the 3758 women invited to participate (Heude et al., 2016). Nested in the wider cohort, a subset of 871 dyads were included in this study, corresponding to the dyads with a cord serum sample and at least one Strength and Difficulties Questionnaire (SDQ) measure filled out at age 3 or 5 (eFig. 1).

### 2.2 Ethics

The EDEN cohort was approved by the ethics committee of Bicêtre Hospital and by the French national committee for data privacy. The written informed consent of both parents was collected for themselves and for their child at his/her birth.

### 2.3 Cytokines in cord serum

Levels of Chemokine (C–C motif) ligand (CCL) 2, CCL3, CCL4, CCL11, CCL13, CCL17, CCL26, C-X-C motif chemokine 10 (CXCL10), Granulocyte-Macrophage Colony-Stimulating Factor (GM-CSF), Vascular Endothelial Growth Factor (VEGF), IFN-γ, IL-1α, IL-1β, IL-2, IL-4, IL-5, IL-6, IL-7, IL-8, IL-10, IL-12p40, IL-12p70, IL-13, IL-15, IL-16, IL-17A, TNF-α and TNF-β were measured in 871 venous cord blood samples obtained immediately after delivery; and allowed to clot for 1 h at room temperature prior to centrifugation (1500×*g*, 10 min). Serum was collected, aliquoted and stored at −80 °C until analysis. All immunoassays were completed within a 4-week period by the same investigator blind to the class of the samples. Concentration of cytokines were measured using multiplex sandwich immunoassays Proinflammatory Panel 1, Cytokine Panel 1 and Chemokine Panel 1 (Meso Scale Diagnostics, Rockvill, USA). Briefly, samples were loaded onto a pre-coated plate with capture antibodies. Commercial buffers were added to create an environment conducive to electrochemiluminescence. The plate was subjected to a voltage allowing light emission on the Sector Imager 2400 plate reader (Meso Scale Diagnostics, Rockvill, USA), the signal was proportional to the amount of the analyte. GM-CSF and VEGF were excluded since they are hematopoietic growth factors. Cytokines with values below detection level in more than 10% of samples were excluded. Among the remaining cytokines, values below detection level were imputed by half of the lower limit of detection of the cytokine.

### 2.4 Mood dysregulation trajectories between the age of 3 and 8 years

Parents completed the French version of the SDQ at the age of 3, 5 and 8 years. The SDQ is a validated questionnaire with 25 items describing 5 dimensions of child behavior: emotional symptoms, conduct problems, hyperactivity/inattention, peer relationship problems and prosocial behavior during the past 6 months (Goodman, 1997, 2001). MD was evaluated through the Strength and Difficulties Questionnaire Dysregulation Profile (SDQ-DP) which includes 15 items of the emotional symptoms (“often complains of headaches, stomach-aches or sickness”, “many worries or often seems worried”, “often unhappy, depressed or tearful”, “nervous or clingy in new situations, easily loses confidence”, “many fears, easily scared”), conduct problems (“often loses temper”, “generally well behaved, usually does what adults request” (reverse score), “often fights with other children or bullies them”, “often lies or cheats”, “steals from home, school or elsewhere”) and hyperactivity (“restless, overactive, cannot stay still for long”, “constantly fidgeting or squirming”, “easily distracted, concentration wanders”, “thinks things out before acting” (reverse score),”good attention span, sees chores or homework through to the end” (reverse score)) dimensions, each item being scored between 0 and 2. The total SDQ-DP subscore ranges from 0 to 30. This validated subscore reflects a dysregulated behavior syndrome which represents deficits of self-regulation and is not specific to a particular psychiatric disorder. The higher the score, the more the child presents a MD profile (Deutz et al., 2018; Holtmann et al., 2011; Winsper and Wolke, 2014).

### 2.5 Covariates

Socio-demographic and medical data related to the parents and the child were collected from pregnancy onwards until 8 years of age through their medical file, physical exams, and self-completed questionnaires. Medical history, socio-economic status and maternal lifestyle were obtained at study inclusion during a standardized interview with a midwife. The mother's mood during her 6th month of pregnancy was evaluated with the Centre of Epidemiological Studies Depression Scale (CES-D) (Radloff, 1977).

### 2.6 Data analysis

#### 2.6.1 Trajectory modelling

MD trajectories between the age of 3 and 8 years were estimated with latent class mixed models (lcmm) for nonlinear outcomes (Proust-Lima et al., 2017; Boucquemont et al., 2017) without any covariates and considering the time variable as linear. Changes in the SDQ-DP score were modeled using quadratic splines with five internal knots. Five different models were tested ranging from one to five classes shown in eTable1. The best model was chosen according to both its entropy and its clinical relevance in terms of prevalence of MD in the general pediatric population (Copeland et al., 2013). According to the model retained, the highest class was considered as pathological whereas the other classes were merged as physiological variations (Dougherty et al., 2016; Vergunst et al., 2019).

#### 2.6.2 Network analysis

Cytokine serum concentrations were standardized and normalized using nonparanormal transformations (Zhao et al., 2012). Mixed graphical models for cytokines and MD trajectories were estimated based on partial correlations (Haslbeck and Waldorp, 2018). LASSO regularization regression was used to identify the most relevant edges among a large number of pairwise associations. The extended Bayesian Information Criterion for which the parameter gamma was set to 0.25 was used to select the LASSO penalty parameter. The edges represented the partial correlations between two nodes of the network after controlling for all the other network variables. One hundred non-parametric bootstraps of the edge weights of non-zero estimate proportions assessed the stability and the accuracy of the network, whereas the strength centrality index assessed the strength of the internode connections.

#### 2.6.3 Selection of cytokines

Owing to the collinearity of cytokines, an elastic net penalized logistic regression was used to select the cytokines of interest. Cytokine concentrations were standardized and log-transformed to be coherently modeled together and to maintain their linearity with the outcome. To obtain the optimal tuning hyperparameters of the model, we used 10-block cross-validation (Genser et al., 2007). As the elastic net is not a deterministic method, we repeated it 1000 times and kept in the final model only cytokines with a stringent variable inclusion probability (VIP) higher than 70%.

#### 2.6.4 Selection of covariates other than cytokines

The covariates for the multivariate analysis were selected with a directed acyclic graph (DAG) after a brief literature review (eFig. 2). Maternal pre-pregnancy body mass index (BMI) predicts neurodevelopmental outcome at the age of 2 (Hinkle et al., 2012) and modifies cord serum levels of inflammatory markers such as CRP (McCloskey et al., 2018; Ross et al., 2022). Smoking during pregnancy is associated with the emergence of neurodevelopmental impairment during childhood and also modifies the placental secretion of some cytokines in vitro (such as TNF-α and IL-6) (Palmer et al., 2016; Munhoz et al., 2017; Belhareth et al., 2018). Alcohol consumption during pregnancy is a well-known risk factor of developing psychiatric disorders during childhood and affects the placental secretion of cytokines (Wilhoit et al., 2017; Ahluwalia et al., 2000). Maternal depression during pregnancy could enhance the offspring's risk of presenting symptoms of MD (Munhoz et al., 2017) and increase blood levels of inflammatory proteins (Lahti-Pulkkinen et al., 2020). Delivery mode has also been linked to an increased risk of developing neurodevelopmental diseases and could also modify the cord serum concentration of cytokines (Zhang et al., 2019; Treviño-Garza et al., 2016). The child's sex is linked to the occurrence of several psychiatric diseases but also can modify the mother's secretion of cytokines (Ross et al., 2022; Mayes et al., 2019; Mitchell et al., 2017).

Considering gestational age at delivery, a recent article shows a potential link between preterm birth and emotional symptoms during childhood (Tsakalidis et al., 2023). It was also found that cytokine dosages in cord blood differed between preterm and term infants (Anderson et al., 2021).

Finally, BMI (non-overweight (<25 kg/m^2^), overweight or obesity (≥25 kg/m^2^)) before pregnancy, smoking (number of cigarettes per day) during pregnancy, drinking alcohol during pregnancy (yes/no), maternal risk for clinical depression at 6 months pregnant (yes (CES-D score≥16)/no (CES-D<16)), delivery mode (vaginal, caesarean or instrumental), the gestational age at delivery and the child's sex (male/female) were selected in the DAG and considered as confounding factors.

#### 2.6.5 Imputation of missing data

No individual in the high mood dysregulation group and 37 (4.3%) individuals in the low mood dysregulation group had missing values in the covariates retained in the main analysis. Except cytokines, missing data were considered as missing at random and were imputed by multivariate imputation using the chained equation method (Buuren and Groothuis-Oudshoorn, 2011). The cytokines selected by the elastic net, the trajectory of MD and all the selected confounders were incorporated into the model. The imputation was repeated 40 times.

#### 2.6.6 Association between cytokines in cord serum and trajectory of mood dysregulation

Logistic regressions were carried out to analyze the association between cord serum cytokines and MD trajectories in a multivariate analysis adjusting for the confounding factors on the imputed data. A sensitivity analysis was performed with univariate analysis, and multivariate analysis was applied to the imputed and the complete data. Weighted (by the lcmm post probabilities) logistic regression was performed to account for the uncertainty of classification of each trajectory class for each of the models. In addition, to test the robustness of the results by considering a different way to model MD trajectories, we conducted a group-based trajectory modeling (GBTM) (Nagin, 2005). Finally, to test whether the results were sensitive to the statistical tools used, we averaged the SDQ DP score over the 3 waves (3, 5, 8 years), instead of using trajectories. Notably, we tested the interaction between the cytokines selected by the elastic net and several covariates (e.g. sex of the newborn, maternal CES-D score during pregnancy, and delivery mode) which could have played a moderating effect.

Statistical analyses were carried out with the statistical software R (version 3.6.1)

## 3 Results

### 3.1 Descriptive characteristics

Of the 871 mother-child dyads included in the study sample, 462 children were males (53%) and 409 were females (47%). The sample's characteristics compared to the rest of the cohort are displayed in eTable 2.

2.07% of the children presented a high MD trajectory between the age of 3 and 8. The mean MD trajectories are shown in eFig. 3 and the posterior probabilities for each class and for the regrouped class are shown in eTable 3 and eTable 4.

Table 1 shows the characteristics of the study sample by child MD trajectory and Table 2 the mean concentration of the cytokines according to MD trajectory. Compared to children with low MD, children with high MD belonged more frequently to families presenting a lower level of parental education, their mothers were more at risk for clinical depression at 6 months pregnant, and they were more frequently male. Moreover, they presented higher MD scores at 3, 5 and 8 years and lower levels of IFN-γ, IL-8 and TNF-α in cord blood.

**Table 1:** Study population characteristics depending on mood dysregulation trajectory (N = 871), EDEN cohort, 2003–2014.

Variables	High mood dysregulation			Low mood dysregulation			p-value^b^
	(N = 18)			(N = 853)			
	n(%)	M (s)	md^c^	n(%)	M (s)	md^c^	
**Parental socio-demographic characteristics**							
Maternal age at birth (years)		28.7 (5.0)	0		30.2 (4.7)	0	0.20
Paternal age at birth (years)		32.2 (5.5)	0		32.4 (5.7)	7	0.92
Couple's monthly income (euros)			0			4	0.63
=<800	1 (5.6)			23 (2.8)			
[801–1500]	2 (11.1)			70 (8.2)			
[1501–3000]	13 (72.2)			498 (58.7)			
[3001–4500]	2 (11.1)			228 (26.8)			
>4500	0 (0)			30 (3.5)			
Parental educational level (years)		12.7 (2.03)	2		13.8 (2.3)	64	0.05
Maternal pre-pregnancy BMI (kg/m^2^)		22.5 (5.3)	0		23.18 (4.3)	14	0.48
Non over-weight (<25)	15 (83.3)			624 (74.4)			0.58
Over-weight (≥25)	3 (16.7)			215 (25.6)			
**Pregnancy characteristics**							
Smoking (cigarettes per day)			0			20	0.41
Yes	5 (27.8)	7.7 (6.2)		170 (20.4)	4.8 (3.9)		0.30
No	13 (72.2)	–		663 (79.6)	–		
Alcohol consumption during pregnancy			0			0	0.64
Yes	9 (50.0)			379 (44.4)			
No	9 (50.0)			474 (55.6)			
CES-D Score^a^ at 6 months pregnant		16.2 (6.0)	0		10.7 (7.7)	4	0.003
Low risk for clinical depression (<16)	10 (55.6)			667 (78.6)			0.04
At risk of clinical depression (≥16)	8 (44.4)			182 (21.4)			
Delivery mode			0			1	0.44
Vaginal	13 (72.2)			656 (77.0)			
Instrumental	1 (5.6)			91 (10.7)			
Caesarean	4 (22.2)			105 (12.3)			
**Child characteristics**							
Sex			0			0	0.03
Male	14 (77.8)			448 (52.5)			
Female	4 (22.2)			405 (47.5)			
Gestational age (amenorrhea weeks)		39.8 (1.5)	0		39.3 (1.6)	0	0.15
Birth weight (grammes)		3398.1 (566.0)	0		3314.3 (488.1)	0	0.47
Mood dysregulation Score							
3 years old		15 (3.0)	0		8.1 (4.0)	78	<0.001
5 years old		19.6 (3.3)	0		7.2 (4.2)	2	<0.001
8 years old		19.2 (2.9)	4		6.8 (3.9)	226	<0.001

c CES-D: Center for Epidemiologic Studies-Depression.

b according to *t*-test of Student for quantitative variables and to χ^2^ and Fisher's test for qualitative variables.

a Md: number of missing data.

**Table 2:** Cord serum cytokines concentration depending on mood dysregulation trajectory (N = 871), EDEN cohort, 2003–2014.

Cytokines (pg/mL)	High mood dysregulation		Low mood dysregulation		p-value*
	(N = 18)		(N = 853)		
	M	(s)	M	(s)	
CCL2	334.1	(75.8)	330.3	(251.8)	0.85
CCL3	20.8	(12.5)	34.5	(161.6)	0.03
CCL4	222.7	(18.0)	266.4	(133.4)	0.02
CCL11	225.9	(76.8)	223.0	(93.9)	0.89
CCL17	1202.5	(754.6)	1480.3	(1098.0)	0.29
CCL26	12.8	(6.1)	13.1	(16.4)	0.85
CXCL10	144.1	(73.2)	204.9	(449.7)	0.01
IFN γ	2.1	(0.8)	2.8	(3.3)	0.002
IL-1b	0.5	(0.4)	1.1	(5.2)	0.003
IL-6	6.1	(4.9)	25.2	(179.3)	0.002
IL-7	8.1	(2.0)	9.3	(3.9)	0.03
IL-8	17.0	(9.2)	59.6	(333.6)	<0.001*
IL-10	0.9	(0.7)	1.2	(4.6)	0.24
IL-12p40	627.5	(210.5)	657.1	(250.7)	0.62
IL-15	2.2	(0.8)	2.8	(7.3)	0.07
IL-16	407.0	(360.3)	449.4	(580.1)	0.63
IL-17	9.7	(7.0)	9.6	(6.5)	0.95
TNF-α	2.7	(0.8)	3.6	(2.5)	<0.001*
TNF-β	0.7	(0.3)	0.8	(0.6)	0.17

*According to the *t*-test of student using a Bonferroni correction: p-value is significant if < 0.002 to take into account multiple testing.

**Table 3:** Adjusted association between cord blood TNF-α concentration (pg/mL) at birth and mood dysregulation trajectories between 3 and 8 on imputed data, EDEN cohort, France, 2003–2014.

Factors	OR	[95%CI]
**Covariates**		
Pre-pregnancy BMI (overweight-obesity vs normal)	0.51	[0.14–1.88]
Maternal risk of clinical depression at 6 months pregnant (yes vs no)	1.07	[1.02–1.13]
Smoking during pregnancy (cigarettes/day)	1.12	[0.99–1.26]
Alcohol during pregnancy (yes vs. no)	1.25	[0.46–3.35]
Gestational age at birth (amenorrhea weeks)	1.31	[0.89–1.94]
Delivery Mode		
Vaginal	Ref	–
Instrumental	0.46	[0.05–3.79]
Ceasarian	1.46	[0.41–5.21]
Child sex (female vs. male)	0.24	[0.07–0.81]
**Cord Serum cytokines**		
TNF-α (pg/mL)	0.35	[0.18–0.67]

### 3.2 Network of cord serum cytokines and mood dysregulation in offspring

**Fig. 1:**
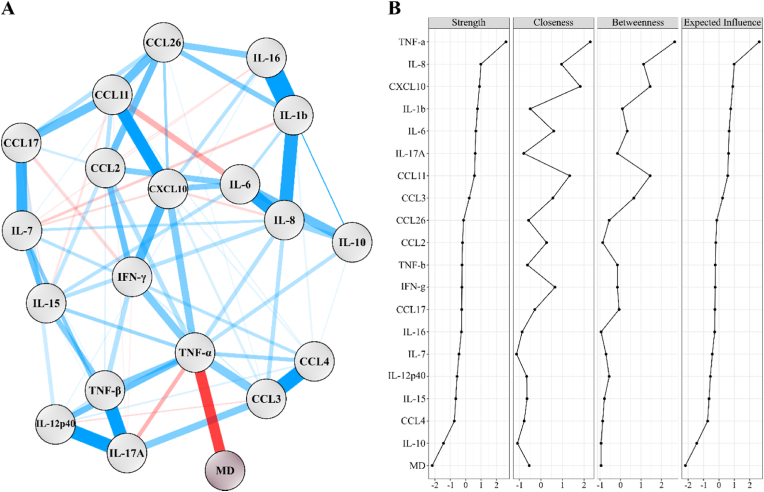
A. Network displaying relationship between 19 standardized cord serum cytokines levels and mood dysregulation trajectories between the age 3 and 8 (MD), N = 871. Blue edges indicate positive relationships and red negative relationships. The absolute value of the association edge-parameter is proportional to the width of the edges. IFN: interferon, IL: interleukin, TNF: tumor necrosis factor. B. computed centrality indices for estimated network based on 19 standardized cord serum cytokines and mood dysregulation trajectories between the age 3 and 8.

Fig. 1 displays the network of 19 cord serum cytokines and MD trajectories with the node strength centrality indices for the 20 nodes. Non-parametric bootstraps on edge weight are shown in eFig. 4.

TNF-α was the most central and most influential cytokine followed by IL-8, CXCL10, IL-1β, IL-6 and IL-17A. Interestingly, TNF-α was the only cytokine negatively related to the high MD trajectory, with a regularized edge weight equal to - 0.25. TNF α was also negatively related to IL-17A and positively related to CXCL10, IFNγ, CCL2, CCL3, CCL4, IL-10, IL12p40 and IL-15.

### 3.3 Longitudinal multivariate associations between cord serum cytokines and MD in offspring

eFig. 5 shows a high degree of correlation between cytokine pairs. We therefore used elastic net penalized regression to select the main cytokines of interest. The elastic net method allowed the selection of one major cytokine of interest: TNF-α with a VIP of 77.2% (eTable 5).

Table 3 shows the association between TNF-α and MD trajectories during childhood in our models. The high MD trajectory between the age of 3 and 8 was negatively associated with TNF-α cord serum levels (adjusted Odds Ratio (aOR) = 0.35, 95%CI [0.18–0.67]), adjusting on the mother's pre-pregnancy BMI category, maternal risk of clinical depression at 6 months pregnant, smoking and alcohol consumption during pregnancy, delivery mode, gestational age at birth and the child's sex on imputed data. Child's sex (Female) was negatively associated with MD trajectories (aOR = 0.24, 95%CI [0.07–0.81]), whereas maternal risk of clinical depression at 6 months pregnant was positively associated with them (aOR = 1.07, 95%CI [1.02–1.13]).

Sensitivity analyses using GBTM to independently model trajectories with a similar inclusion rate as the main analysis (29 (3.3%) out of the 871 offsprings were classified in the high mood dysregulation trajectory) and when considering the average SDQ DP score, showed the same pattern of results (eTable 6). Other confounders (such as maternal uro-genital infection during pregnancy and maternal medical history of allergies) were not maintained after inclusion in the DAG. However, considering their clinical importance, we chose to run a sensitivity analysis, that reassuringly found the same pattern of results (aOR = 0.35 95%CI [0.17–0.68]).

## 4 Discussion

### 4.1 Principal findings

A lower cord serum concentration of TNF-α at birth was associated with a pathological MD trajectory during childhood. In our study, TNF-α appeared to be both the most central and the most influential cytokine at birth with regard to pathological MD trajectory, when considering a large network of 19 cord cytokines. Notably, TNF-α was at the crossroads of interaction between multiple cytokines, thus illustrating the complex cascade of their interrelationships. This finding linking TNF-α at birth to child MD adds to the available literature relating blood levels of TNF-α and psychiatric disorders.

On one hand, several studies relying on adult cross-sectional samples have shown positive associations between TNF-α and depression (Dowlati et al., 2010; Kokkosis and Tsirka, 2020; Hoseth et al., 2017). A network study, showed that TNF-α was the core inflammatory cytokine in adult patients presenting depressive symptoms with severe retardation and cognitive disorders (Zeng et al., 2023).On the other hand, studies focusing on a range of specific situations have documented negative associations between various psychiatric disorders and TNF-α. First, contrary to adults, low blood levels of TNF-α were found both in adolescents with severe depression and suicidal thoughts (Gabbay et al., 2009a) and in children with dysthymia (Brambilla et al., 2004). Second, a negative correlation was also found between blood levels of TNF-α and the severity of treatment-resistant schizophrenia patients (Mostaid et al., 2018). Third, an elevation in TNF-α blood levels in women during the perinatal period had a protective effect on the emergence of postpartum depression (Buglione-Corbett et al., 2018; Corwin et al., 2015). Most importantly in the context of our current findings, a study by Gilman et al. (2016) found that high maternal TNF-α during the third trimester of pregnancy was also associated with reduced risk of depression in their offspring in adulthood. Interestingly, while psychiatric side-effects are well known with anti-TNF-α drug treatments (Thillard et al., 2020), these same drugs can improve depressive symptoms but only in patients suffering from depression with high levels of peripheral inflammatory markers (Raison et al., 2013).

Interestingly, our descriptive analyses also found a statistical difference of IL-8 between the two MD groups. It has been shown that higher levels of IL-8 are protective against onset of depression and are related to less severe depressive and anxious symptoms among patients (Skibinska et al., 2022; Kruse et al., 2022).

Moreover, cytokine expression fluctuates through different stages of cortical human development during infancy and adolescence (Sager et al., 2023). Altogether, these findings suggest that the role of TNF-α might vary according to factors such as developmental windows and brain region.

### 4.2 Interpretation

*In vitro* and animal studies may also shed light on our findings. TNF-α can play different roles depending on its cellular source of secretion, its cellular targets, and presumably the window of neurodevelopment during which it acts. In the developing brain, NPC, neurons, astrocytes and microglia express TNF-α and its receptors or cytokine receptors (Deverman and Patterson, 2009). Animal studies have shown that peripheral TNF-α can cross the blood-brain barrier to act directly on the brain parenchyma (Banks et al., 1995). *In vitro*, TNF-α induces a shift from the neuronal to the astroglial lineage in differentiating NPC (Johansson et al., 2008), likely by increasing the apoptosis of NPC-derived neurons (Wang et al., 2011). Animal studies support a role for TNF-α in neurodevelopment the balance of excitatory/inhibitory synapses formation (Wei et al., 2012), hippocampal maturation (Golan et al., 2004), and synaptic scaling (Stellwagen and Malenka, 2006). These data suggest that TNF-α signaling around birth impacts neurodevelopment and possibly later behavioral outcome. An elevation of this cytokine in mice is associated with learning and memory difficulties (Park and Bowers, 2010). *In utero*, exposure to elevated concentrations of TNF-α is associated with an increase in anxiety and depressive-like symptoms in male mice offspring, whereas it was found to protect against anxiety in female offspring (Babri et al., 2014). Interestingly, the overexpression of pro- or anti-inflammatory cytokines in pregnant rats was associated with cognitive and behavioral impairments in the offspring (Ratnayake et al., 2013). This strengthens the notion that neurodevelopmental pathologies could depend on an immune imbalance and that the role of TNF-α could differ according to the gestational window.

### 4.3 Strengths and weaknesses

This study has several limitations. First, the study population was more educated, had higher socio-economic levels, mothers had lower depression scores during pregnancy and their babies had a higher birth weight than the rest of the EDEN cohort. Therefore, we may have introduced a selection bias towards a less severe subsample. This may have led to under-estimating the force of the association between TNF-α and the pathological trajectories of MD. Second, a measurement bias cannot be excluded concerning the SDQ scores, since it exclusively relied on a parental report and did not include reports from other caregivers or teachers. Of note, the interindividual heterogeneity was sparse since MD symptoms were assessed at only three different timepoints. Third, the limited number of individuals presenting the MD pathological trajectory in our study could make our results unstable, although they were confirmed in several sensitivity analyses, including when widening the high MD trajectory class. Nevertheless, the proportion of children presenting MD symptoms in the study was similar to the proportion of children displaying high-level MD symptoms in the general population (Copeland et al., 2013). Fourth, since TNF-α is known to play a homeostatic role in labor and childbirth (Peltier, 2003), it might merely represent a proxy of the fetal state at birth. Reassuringly, however, we found no interaction between TNF-α and delivery mode or other perinatal variables, thereby arguing against this limitation. Fifth, A potential weakness of measuring biomarkers in the cord blood at birth is that it may not reflect immune activity at the relevant time point in fetal neurodevelopment. Finally, our findings could be linked to residual confounders or reflect unknown physiological mechanisms.

The innovative feature of our work is a major strength of this study. To our knowledge, it is the only prospective longitudinal study to focus on the association between several cord serum cytokines and MD trajectories during early childhood. The design with a follow-up period going from the second trimester of pregnancy to the age of 8 years, the prospective data collection through medical records and the assessment of MD through a standardized questionnaire commonly used in epidemiological studies are methodological strengths. Finally, we combined state-of-the-art statistical methods (trajectory modeling, penalized regression, network analysis) to depict MD trajectories and to select the cytokines of interest accounting for their collinearity.

### 4.4 Unanswered questions and further research

Although promising, these findings should be interpreted with caution when considering their possible implications. In terms of mechanistic understanding, they support the hypothesis that immune activity during the perinatal period is a critical factor for the future neurodevelopment of youth. Particularly, perinatal cytokine balance may play an important role in the early emergence of transnosographic psychiatric symptoms.

Linking a biomarker to a given clinical psychiatric dimension, as advocated by the RDoC, appears useful to reach a better understanding of its etiological contribution and may open perspectives on preventive interventions. Nevertheless, these explorations are in their infancy and we are a long way from being able to predict neurodevelopment in humans or treat deficiencies. However, animal investigations should be conducted. Animal models could serve to test whether variations in TNF-α drive neurodevelopmental outcomes in the offspring by using TNF-α and anti-TNF-α molecules during late gestation or in the perinatal period to intervene on TNF-α exposure. At a human epidemiological level, it could be relevant to follow children's psychiatric outcome in a birth cohort comprising mothers treated by anti-TNF-α molecules.

To conclude, the protective effect of TNF-α at birth against the occurrence of MD trajectories during childhood is a finding that offers much promise for the future. The putative physiopathological mechanisms underlying the development of MD may now be explored in a fresh perspective.

## Funding

This study was supported by the 10.13039/501100004431Fondation de France, Fondation pour la Recherche Medicale, the French Ministry of Research Institut Federatif de Recherche and Cohort Program, the INSERM Nutrition Research Program, the French Ministry of Health Perinatal Program, the French Agency for Environmental Safety, the French National Institute for Population Health Surveillance, 10.13039/501100007486Paris-Sud University, the French National Institute for Health Education, Nestle, Mutuelle Generale de l’Education Nationale, the French-Speaking Association for the Study of Diabetes and Metabolism (Alfediam), the National Agency for Research (10.13039/501100001665French National Research Agency non-thematic program), the National Institute for Research in Public Health (Institut de Recherche en Sante Publique, the Tres Grande Infras-tructure de Recherche Cohorte Sante, 2008 Program), and the 10.13039/501100001665French National Research Agency Grants No. ANR-12-DSSA-0005-01 and No. ANR-23-CE36-0003-01. Marie Herbein received a 10.13039/501100008312Pierre Deniker Foundation grant.

## CRediT authorship contribution statement

**Marie Herbein:** Formal analysis, Writing – original draft, Writing – review & editing. **Susana Barbosa:** Writing – review & editing. **Ophélie Collet:** Writing – review & editing. **Olfa Khalfallah:** Data curation, Writing – review & editing. **Marie Navarro:** Writing – review & editing. **Marion Bailhache:** Writing – review & editing. **Nicolas IV:** Writing – review & editing. **Bruno Aouizerate:** Writing – review & editing. **Anne-Laure Sutter-Dallay:** Writing – review & editing. **Muriel Koehl:** Writing – review & editing. **Lucile Capuron:** Writing – review & editing. **Pierre Ellul:** Writing – review & editing. **Hugo Peyre:** Writing – review & editing. **Judith Van der Waerden:** Writing – review & editing. **Maria Melchior:** Writing – review & editing. **Sylvana Côté:** Writing – review & editing. **Barbara Heude:** Methodology, Writing – review & editing, Conceptualization. **Nicolas Glaichenhaus:** Writing – review & editing. **Laetitia Davidovic:** Conceptualization, Methodology, Writing – review & editing. **Cedric Galera:** Conceptualization, Methodology, Supervision, Writing – original draft, Writing – review & editing.

## Declaration of competing interest

We declare none of the authors has any conflict of interest regarding the manuscript “Cord serum cytokines at birth and children's trajectories of mood dysregulation symptoms from 3 to 8 years: the EDEN birth cohort”

## Data Availability

no direct acces to data please contact correspondant author
