# Supplementary material for: Cord serum cytokines at birth and children's trajectories of mood dysregulation symptoms from 3 to 8 years: The EDEN birth cohort
Source: Brain Behav Immun Health. 2024 Mar 29;38:100768. doi: 10.1016/j.bbih.2024.100768 (PMC10990861; doi:10.1016/j.bbih.2024.100768)
Supplement: Multimedia component 1 [file mmc1.docx]

| ***eTable 1. Latent Class Mixed models estimations*** | | | | | | | | | | | | |
| --- | --- | --- | --- | --- | --- | --- | --- | --- | --- | --- | --- | --- |
| Model | Grp^1^ | Loglik^2^ | npm^3^ | BIC^4^ | Entropy | Conv^5^ | AIC^6^ | %cl1^7^  (n)^8^  *pprob^9^* | %cl2^7^  (n)^8^  *pprob^9^* | %cl3^7^  (n)^8^  *pprob^9^* | %cl4^7^  (n)^8^  *pprob^9^* | graph |
| M1b | 1 | -6138.2 | 11 | 12350.8 | 1 | yes | 12298.3 | __ | _ | _ | _ | _ |
| M2b | 2 | -6134.5 | 15 | 12370.6 | 0.53 | yes | 12299.1 | 12.3  (107)  *0.72* | 87.7  (764)  *0.88* | _ | _ | 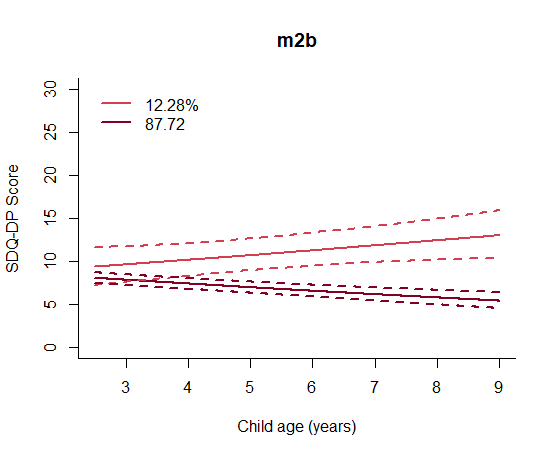 |
| M3b | 3 | -6132.3 | 19 | 12393.2 | 0.42 | yes | 12302.6 | 8.6  (75)  *0.70* | 47.8  (416)  *0.67* | 43.6  (380)  *0.75* | _ | 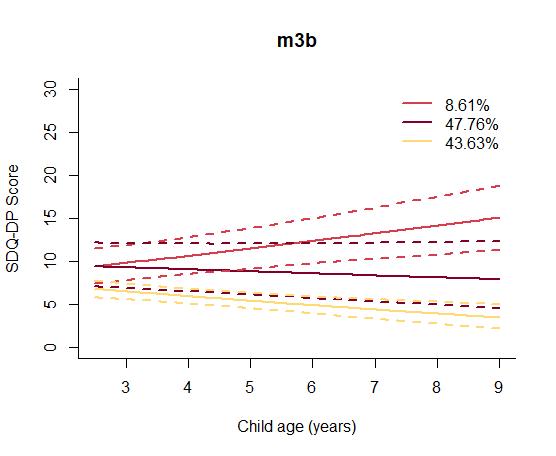 |
| M4b | 4 | -6129.8 | 23 | 12415.3 | 0.63 | yes | 12305.6 | 2.1  (18)  *0.76* | 7.8  (68)  *0.72* | 36.6  (319)  *0.79* | 53.5  (466)  *0.77* | 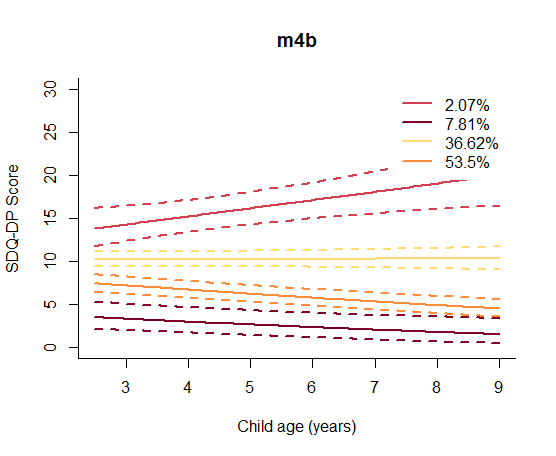 |
| M5b | 5 | -6129.5 | 27 | 12441.8 | 0.34 | no | 12313.0 | _ | _ | _ | _ | _ |
| ^1^ number of groups ; ^2^Loglikelihood ; ^3^ number of parameters, ^4^Bayesian Inclusion Criteria, ^5^Convergence, ^6^Akaike Criteria, ^7^class percentage, ^8^number of subjects, ^9^postprobability to belong to the class | | | | | | | | | | | | |

*eTable2. Characteristics of the dyads included in the study and the rest of the cohort, EDEN cohort, France, 2003-2014.*

| Variables | Dyads included | | Dyads of the rest of the cohort | |  |
| --- | --- | --- | --- | --- | --- |
|  | (N=871) | | (N=1131) | | p-value° |
|  | N (%) | M (s) | N (%) | M (s) |  |
| **Parental socio-demographic characteristics** | | | | | |
| Maternal age at birth (years) | 871 | 30.1 (4.7) | 1036 | 28.9 (5.0) | <0.001 |
| Paternal age at birth (years) | 864 | 32.4 (5.7) | 1037 | 31.5 (6.1) | 0.001 |
| Couple’s monthly income (euros)     =<800     [801-1500]     [1501-3000]     [3001-4500]     >4500 | 867  24 (2.7)  72 (8.3)  511 (59)  230 (26.6)  30 (3.5) |  | 1046  69 (6.6)  162 (15.5)  558 (53.3)  212 (20.3)  45 (4.3) |  | <0.001 |
| Parental educational level (years) | 805 | 13.8 (2.3) | 890 | 13.1 (2.3) | <0.001 |
| Maternal pre-pregnancy BMI (kg/m²) | 857 | 23.2 (4.4) | 1027 | 23.3 (4.8) | 0.50 |
| Non over-weight (<25)    Over-weight (≥ 25) | 639 (74.6)  218 (25.4) |  | 749 (72.9)  278 (27.1) |  | 0.42 |
| **Pregnancy characteristics** |  |  |  |  |  |
| Smoking (cigarettes per day)     Yes     No | 851  175 (20.6)  676 (79.4) | 4.9 (4.0)  — | 996  309 (31.0)  687 (69.0) | 6.2 (5.0)  — | <0.001  0.001 |
| Alcohol consumption during pregnancy     Yes     No | 871  388 (44.5)  483 (55.5) |  | 1053  430 (40.8)  623 (59.2) |  | 0.10 |
| CES-D Score* during pregnancy     Non depressed (<16)     Dpressed (≥16) | 867  677 (78.1)  190 (21.9) | 10,8 (7,7) | 1046  755 (72,2)  291 (27,8) | 12,5 (8,4) | <0.001  0,003 |
| Delivery mode     Vaginal     Instrumental     Caesarean | 870  669 (76,9)  92 (10,6)  109 (12,5) |  | 1029  736 (71,5)  103 (10,0)  190 (18,5) |  | 0.002 |
| **Child characteristics** |  |  |  |  |  |
| Sex     Male     Female | 871  462 (53,0)  409 (47,0) |  | 1032  538 (52,1)  494 (47,9) |  | 0.69 |
| Gestational age (amenorrhea weeks) | 871 | 39,3 (1,6) | 1034 | 39,2 (1,9) | 0.10 |
| Birth weight (grammes) | 871 | 3316,1 (489,6) | 1028 | 3247,0 (528,9) | 0.003 |
| Mood dysregulation Score     3 years old     5 years old     8 years old | 871  793  869  641 | 8,3 (4,1)  7,4 (4,6)  7,1 (4,3) | 557  514  315  233 | 8,8 (4,3)  7,9 (4,8)  7,7 (4,8) | 0.03  0.10  0.04 |

** CES-D:* Center for Epidemiologic Studies-Depression

° according to t test of Student for quantitative variables and to χ^2^ and Fisher’s test for qualitative variables

*eTable 3. Mean posterior probabilities to belong to each class, EDEN cohort, France, 2003-2014.*

| Classes | N (%) | Mean posterior probability to belong to each class (%) | | | | |
| --- | --- | --- | --- | --- | --- | --- |
|  |  | Class 1  Highest trajectory | Class 2  Lowest  trajectory | Class 3  Medium high trajectory | Class 4  Medium low trajectory | Total |
| Class 1 | 18 (2,07) | 75,97 | 0 | 23,99 | 0,04 | 100 |
| Class 2 | 68 (7,81) | 0 | 72,46 | 0,48 | 27,06 | 100 |
| Class 3 | 319 (36,62) | 3,41 | 0,02 | 79,31 | 17,26 | 100 |
| Class 4 | 466 (53,5) | 0 | 7,67 | 15,44 | 76,89 | 100 |

*eTable 4. Mean posterior probabilities to belong to either the MD pathological class or to the physiological class, EDEN cohort, France, 2003-2014.*

| Class | N (%) | Mean posterior probability to belong to each class (%) | | |
| --- | --- | --- | --- | --- |
|  |  | High mood dysregulation | Low mood dysregulation | Total |
| High mood dysregulation | 18 (2,07) | 75,97 | 24,03 | 100 |
| Low mood dysregulation | 853 (97,93) | 1,28 | 98,72 | 100 |

*eTable 5. Variable inclusion probability (VIP) for all the cytokines included in the elastic net.*

| Cytokines* | VIP (%) |
| --- | --- |
| CCL2 | 34,4 |
| CCL3 | 37,2 |
| CCL4 | 21,3 |
| CCL11 | 19,2 |
| CCL17 | 19,4 |
| CCL26 | 26,7 |
| CXCL10 | 10,3 |
| IFN γ | 17,0 |
| IL-1b | 20,4 |
| IL-6 | 29,2 |
| IL-7 | 20,1 |
| IL-8 | 8,5 |
| IL-10 | 27,7 |
| IL-12p40 | 15,4 |
| IL-15 | 13 ,3 |
| IL-16 | 14,8 |
| IL-17 | 15,7 |
| TNF-α | 77,2 |
| TNF-β | 13,2 |

*eTable 6. Sensitivity analysis of the association between cord blood TNF-α concentration (pg/mL) at birth and mood dysregulation trajectories between 3 and 8 with different mood dysregulation trajectory modelling, EDEN cohort, France, 2003-2014.*

|  | OR | [95%CI] |
| --- | --- | --- |
| **Mood dysregulation trajectories according to 4 class^a^ Lcmm^b^** |  |  |
| **Logistic regression** |  |  |
| Univariate model | 0.34 | [0.18 – 0.63] |
| Multivariate model^c^ on complete data | 0.30 | [0.15 – 0.58] |
| Multivarate model on imputed data including additional covariates* | 0.35 | [0.17 – 0.68] |
| **Weighted logistic regression^d^** |  |  |
| Univariate model | 0.31 | [0.15 – 0.64] |
| Multivariate model^c^ on complete data | 0.29 | [0.13 – 0.63] |
| Multivariate model^c^ on imputed data | 0.29 | [0.13 – 0.64] |
| **Mood dysregulation trajectories according to GBTM**^e^ |  |  |
| Univariate model | 0.68 | [0.44-1.05] |
| Multivariate model^c^ on complete data | 0.69 | [0.44-1.07] |
| Multivariate model^c^ on imputed data | 0.70 | [0.45-1.09] |
|  |  |  |
| **Linear regression**^f^ | β | [95%CI] |
| Univariate model | -0.10 | [-0.002 – 0.21] |
| Multivariate model^c^ on complete data (all waves) | -0.11 | [-0.005 – 0.21] |
| Multivariate model^c^ on complete data (wave 3 years) | -0.08 | [-0.22 – 0.05] |
| Multivariate model^c^ on complete data (wave 5 years) | -0.13 | [-0.25 – -0.005] |
| Multivariate model^c^ on complete data (wave 8 years) | -0.13 | [-0.33 – 0.07] |
| Multivariate model^c^ on imputed data (all waves) | -0.11 | [-0.005 – 0.21] |

*^a^high mood dysregulation n=18 (2.07%)*

*^b^latent class mixed model*

*^c^ adjusted on the mother’s pre-pregnancy BMI category, mother depression during pregnancy, smoking and drinking alcohol during pregnancy, delivery mode and the child’s sex.*

*^d^weighted logistic regression considering the lcmm classification of mood dysregulation trajectories uncertainty ^e^grouped based-trajectory model, 5 classes, high mood dysregulation n= 29 (3,3%)*

*^f^linear regression considering the SDQ-DP score as a continuous variable*

**adjusting for uro-genital infection during pregnancy and maternal history of allergy*
