# Supplementary material for: Cord serum cytokines at birth and children's trajectories of mood dysregulation symptoms from 3 to 8 years: The EDEN birth cohort
Source: Brain Behav Immun Health. 2024 Mar 29;38:100768. doi: 10.1016/j.bbih.2024.100768 (PMC10990861; doi:10.1016/j.bbih.2024.100768)
Supplement: Multimedia component 2 [file mmc2.docx]

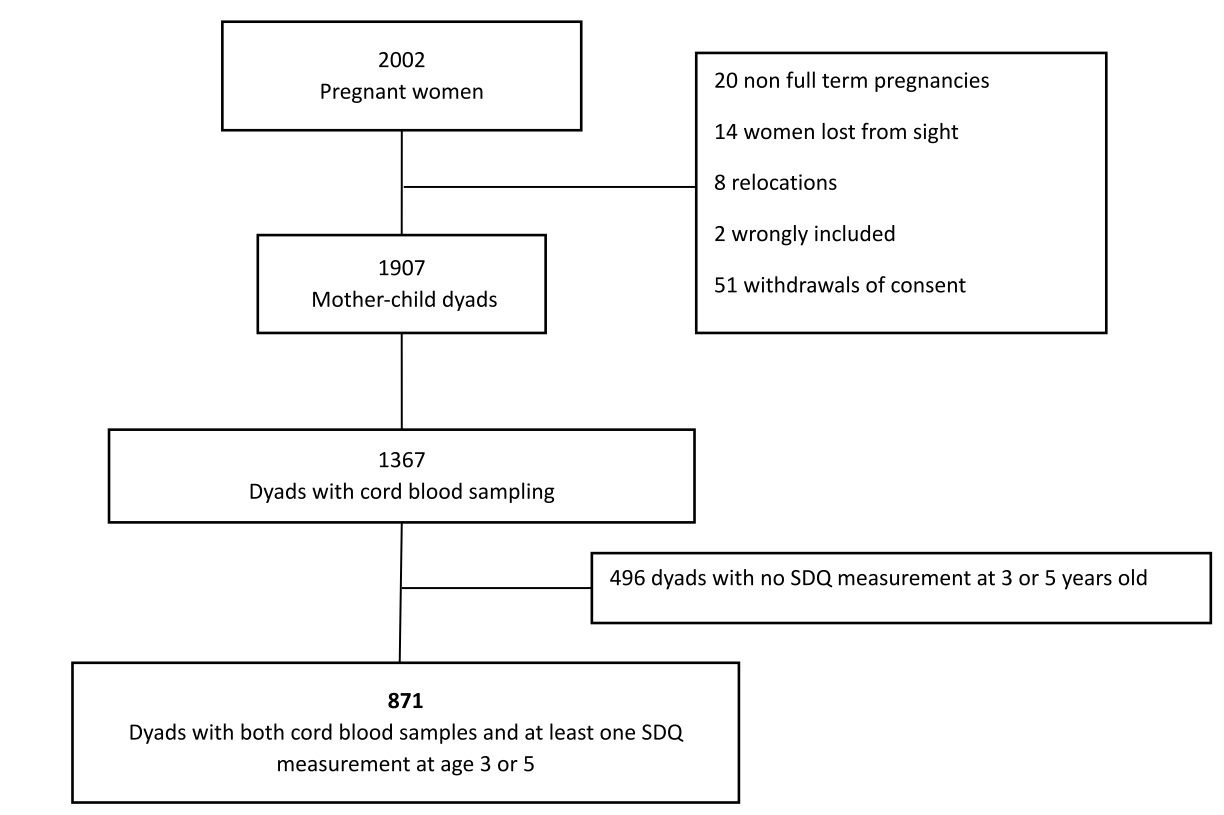


eFigure 1. Flow chart of the nested EDEN cohort, France, 2011.


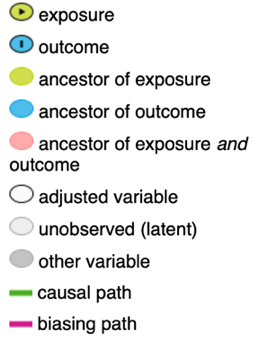

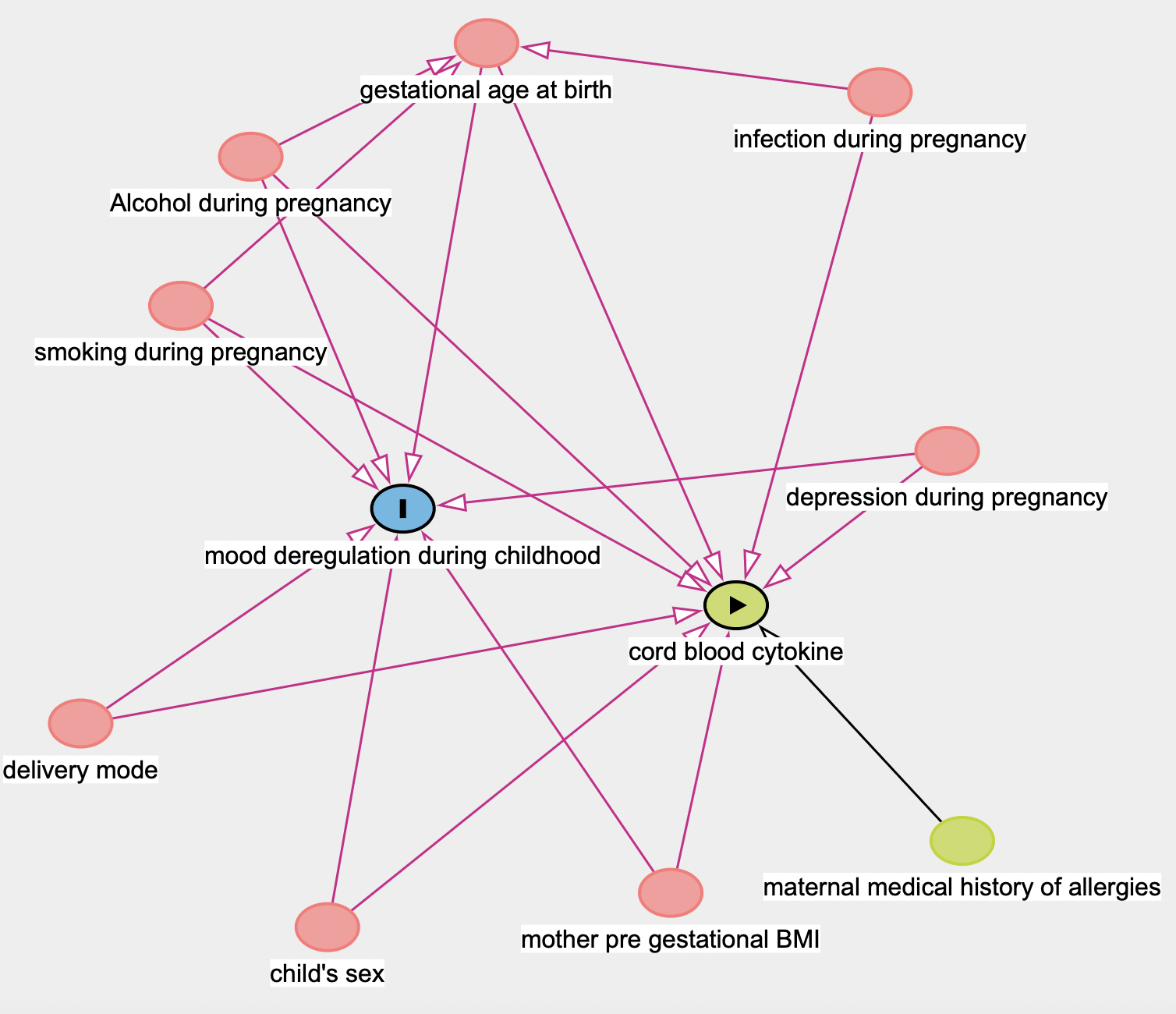


eFigure 2. Directed Acyclic Graph showing the confounding variables on the association between perinatal inflammation and mood dysregulation during childhood.

*BMI : Body Mass Index*


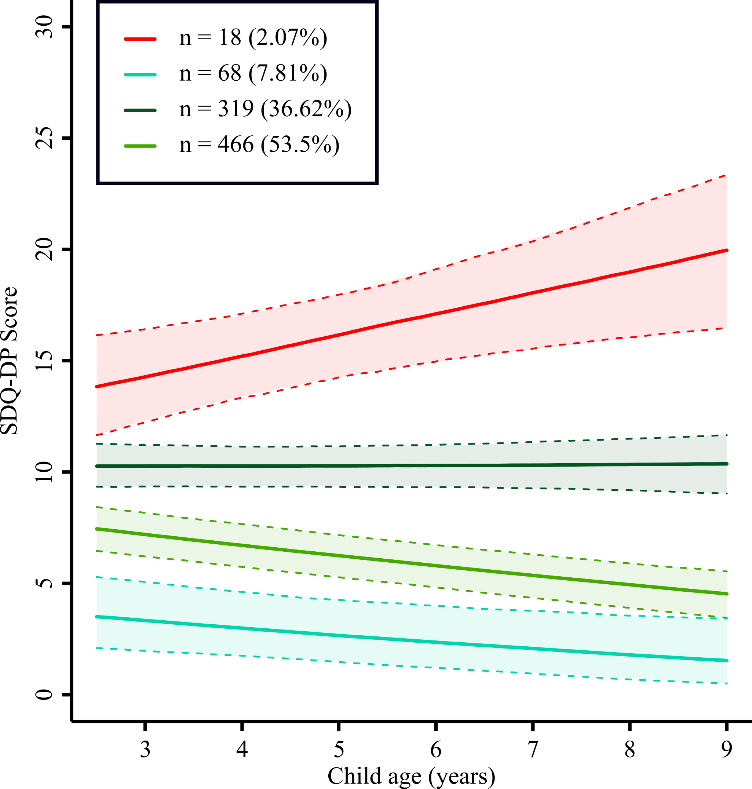


*eFigure 3. Mean SDQ-DP trajectory estimation with its 95%confidence interval (N=871). For each class, n is the number of children in each class (a posteriori), EDEN cohort, France, 2006-2014.*


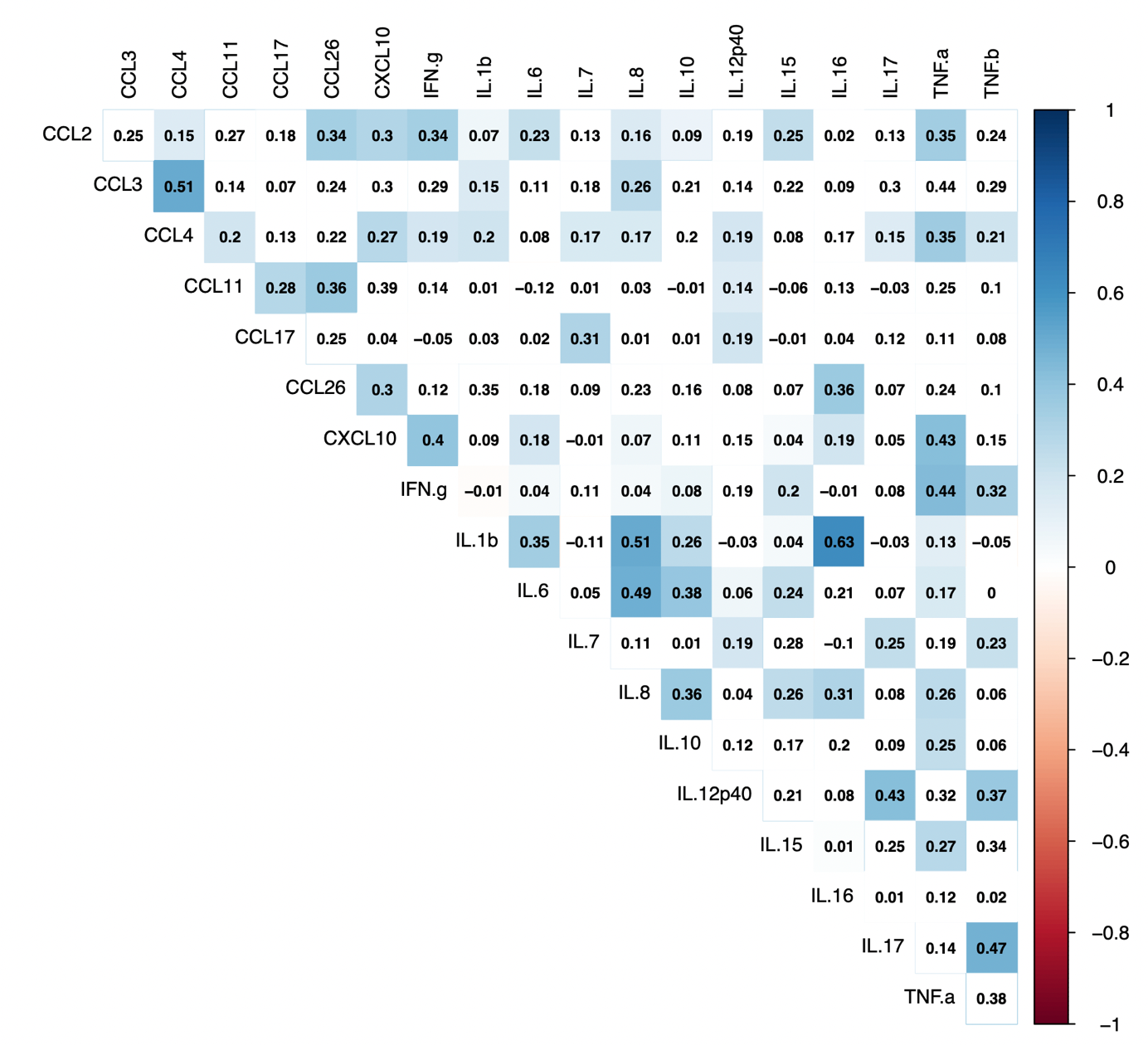


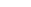


*eFigure 5. Heatmap of the pairwise Spearman’s rank correlation coefficient between all standardized cord serum cytokine pairs*

*Statistical significance of correlation is indicated by background color (it is blank when non-significant) at a significant level of 0.05/171 (Bonferroni correction)*
