# Supplementary figures and images for: Cord serum cytokines at birth and children's trajectories of mood dysregulation symptoms from 3 to 8 years: The EDEN birth cohort
Source: Brain Behav Immun Health. 2024 Mar 29;38:100768. doi: 10.1016/j.bbih.2024.100768 (PMC10990861; doi:10.1016/j.bbih.2024.100768)

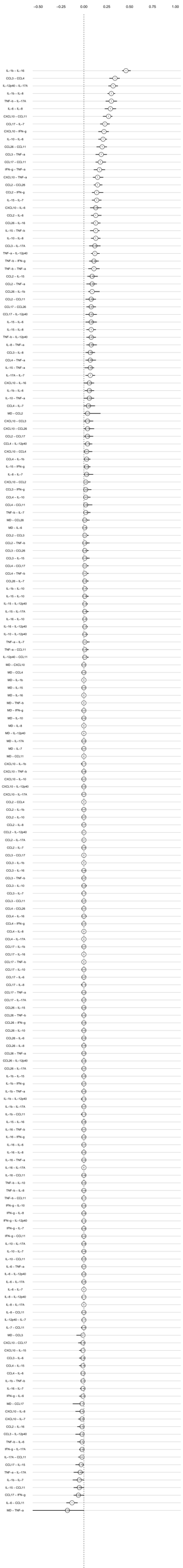

Supplement: Multimedia component 3 [file mmc3.pdf]
